# Methylene Blue Treatment in Refractory Shock Following Chemotherapy: A Case Report

**DOI:** 10.7759/cureus.86559

**Published:** 2025-06-22

**Authors:** Tarana Goyushova, Afaq Ahmadova, Agshin Aliyev

**Affiliations:** 1 Intensive Care Unit, Liv Bona Dea Hospital, Baku, AZE; 2 Critical Care Medicine, Liv Bona Dea Hospital, Baku, AZE

**Keywords:** chemotherapy, crrt, docetaxel, methylene blue, refractory shock, renal failure, vasoplegia, vasopressors

## Abstract

Refractory shock is a life-threatening condition characterized by persistent hypotension and tissue hypoperfusion despite adequate fluid resuscitation and high-dose vasopressors. It frequently progresses to multiorgan failure and has a high mortality rate. We report the case of a 68-year-old male with metastatic prostate adenocarcinoma who developed refractory shock shortly after receiving docetaxel chemotherapy. Despite maximal vasopressor and steroid therapy, the patient remained hypotensive with rising lactate levels. Administration of methylene blue (MB) resulted in rapid hemodynamic improvement, normalization of lactate, and recovery of renal and respiratory function. This case highlights the potential role of MB as an adjunct in managing vasoplegic shock unresponsive to standard therapies.

## Introduction

Refractory shock affects approximately 6%-7% of critically ill patients and is defined by persistent hypotension and end-organ hypoperfusion despite aggressive management with fluid resuscitation and high-dose vasopressors [1,2]. While septic shock is the most common etiology in adults, vasoplegic shock may also occur in the context of chemotherapy, trauma, or systemic inflammation [3]. Vasoplegia involves excessive production of nitric oxide (NO), resulting in profound vasodilation and resistance to catecholamines. When vasopressor doses exceed 1 µg/kg/minute of norepinephrine or equivalent and hypotension persists, mortality may reach up to 80%-90% [4]. Methylene blue (MB) acts as an inhibitor of NO synthase and guanylate cyclase, counteracting the vasodilatory cascade. Its use in vasoplegic syndromes, especially after cardiac surgery, has shown promising results [5-9].

## Case presentation

A 68-year-old male with a known history of metastatic prostate adenocarcinoma (staged T2N1M1) was admitted to the hospital for planned initiation of chemotherapy with docetaxel. Before admission, he had been functionally independent and had not required hospitalization in recent months. Shortly after initiating the infusion, the patient developed acute respiratory distress accompanied by profound hypotension. He was rapidly transferred to the intensive care unit (ICU) for further management. On ICU admission, his blood pressure was 65/40 mmHg, and serum lactate was 8 mmol/L, indicating severe tissue hypoperfusion. The clinical picture was consistent with distributive shock, and a possible chemotherapy-induced anaphylactoid or systemic inflammatory response was considered. Despite aggressive hemodynamic support, including norepinephrine (up to 0.8 µg/kg/minute), epinephrine (up to 0.3 µg/kg/minute), dopamine (10 µg/kg/minute), fluid therapy, and stress-dose corticosteroids (hydrocortisone 200 mg/day), the patient’s mean arterial pressure (MAP) remained <60 mmHg, and lactate rose further to 14.1 mmol/L. Due to progressive respiratory failure, the patient was intubated and placed on mechanical ventilation (bilevel positive airway pressure mode). He also became anuric, prompting the initiation of continuous renal replacement therapy (CRRT) for acute kidney injury and fluid overload. Given the lack of response to conventional therapy, MB was administered as a rescue intervention. The initial dose was 1 mg/kg IV over 30 minutes, and maintenance infusion was 0.5 mg/kg/hour for eight hours. Following MB administration, the patient’s hemodynamics improved significantly. His blood pressure stabilized, increasing from 100/45 mmHg to 110/55 mmHg, and his lactate levels decreased to 2.0 mmol/L (Table 1). Additionally, his urine output resumed, allowing for CRRT to be discontinued after 48 hours. He was successfully extubated on hospital day 14, showing no signs of recurrent hemodynamic instability.

**Table 1:** Blood gases on admission to the ICU and eight hours after the infusion of MB. ICU = intensive care unit; MB = methylene blue

Parameter	Reference range	Before MB	After MB
pH	7.350–7.450	7.098	7.404
pCO₂ (mmHg)	32.0–48.0	31.0	39.2
pO₂ (mmHg)	83.0–108.0	131	109
HCO₃⁻ (mmol/L)	21.2–28.3	10.5	24.3
Base excess (mmol/L)	–3.0–3.0	–20.1	–0.2
Lactate (mmol/L)	0.5–1.6	14.1	2.0
Sodium Na⁺ (mmol/L)	135–146	147	141
Potassium K⁺ (mmol/L)	3.4–4.5	4.0	3.9

## Discussion

The pathophysiology of vasoplegic shock involves systemic inflammatory response, endothelial dysfunction, and overproduction of NO, leading to reduced vascular tone and resistance to vasopressors. MB mitigates these effects by inhibiting NO synthase and guanylate cyclase activity, restoring vascular tone [5]. Although traditionally used in post-cardiac surgery vasoplegia, MB has also been reported in cases of sepsis, liver transplantation, and chemotherapy-induced shock. In our patient, the likely contributors to vasoplegia were docetaxel-induced endothelial activation and systemic inflammatory response. Several case series and observational studies have demonstrated improved MAP, decreased vasopressor requirements, and reduced mortality with MB in vasoplegic states [6-9]. Nevertheless, standardized protocols regarding its use, dosing, and timing remain under development.

## Conclusions

This case highlights the potential efficacy of MB as an adjunctive therapy in managing refractory shock in patients unresponsive to conventional vasopressors. It may be especially valuable in vasoplegic states related to chemotherapy or systemic inflammation. Further research is necessary to validate its routine use and establish clinical guidelines.
